# Are associations between electronic media use and BMI different across levels of physical activity?

**DOI:** 10.1186/s12889-015-1810-6

**Published:** 2015-05-19

**Authors:** Ole Melkevik, Ellen Haug, Mette Rasmussen, Anne Siri Fismen, Bente Wold, Alberto Borraccino, Erik Sigmund, Robert Balazsi, Jens Bucksch, Jo Inchley, Margarida Gaspar de Matos, Oddrun Samdal

**Affiliations:** Division for Mental Health, Norwegian institute of Public Health, Bergen, Norway; NLA University College, Bergen, Norway; National Institute of Public Health, University of Southern Denmark, Odense, Denmark; Department of Health Promotion and Development, University of Bergen, Bergen, Norway; Department of Public Health and Microbiology, University of Turin, Turin, Italy; Center for Kinanthropology Research, Palacky University, Olomouc, Czech Republic; Faculty of Psychology and Educational Sciences, Babes Bolyai University, Cluj-Napoca, Romania; Bielefeld University, School of Public Health, Department of Prevention and Health Promotion, Bielefeld, Germany; Child and Adolescent Health Research Unit, University of St Andrews, Scotland, UK; Faculdade de Motricidade Humana, Universidade de Lisboa, Lisboa, Portugal

## Abstract

**Background:**

The use of electronic media has been found to be a risk factor for higher BMI and for being overweight. Physical activity has been found to be associated with lower BMI and lower risk for being overweight. Little is known about whether the associations between physical activity and electronic media use are additive or interactive in predicting BMI and risk for overweight among adolescents.

**Methods:**

The data used in this study stem from the 2009/2010 survey of “Health Behaviour in School-aged Children (HBSC) study: A WHO Cross-National Survey. The sample consisted of 107184 13 and 15 year students from 30 different countries. Multilevel regression models were used to produce the presented estimates.

**Results:**

Overall, 18% of boys and 11% of girls were classified as overweight. EM use was found to be associated with increased BMI z-scores and odds for overweight among both boys and girls who did not comply with physical activity guidelines. Among physically active adolescents, EM was found to be significantly associated with BMI or odds for overweight among girls, but not among boys.

**Conclusion:**

While the usage of EM appear to be inconsequential for BMI and the risk of overweight among physically active boys, we find evidence indicating that EM use is associated with BMI and risk for overweight among girls, including those who report complying with MVPA guidelines.

## Background

The understanding of modifiable risk factors for overweight and obesity among children and adolescents is of critical importance due to its high prevalence across most parts of the western and developing world. Indeed, a recent review suggests that approximately 30% of American adolescents and 22%–25% of European adolescents are overweight or obese [1]. While some studies indicate that prevalence of overweight and obesity have plateaued in some parts of the world [2-4], prevalence is still high and preventive efforts are required [5].

Overweight and obesity among youth is associated with a range of poor health outcomes including multiple cardiovascular risk factors [6] and non-insulin dependent diabetes mellitus [7]. In addition, obese children (Body Mass Index (BMI) ≥30) in particular have been found to have lower health related quality of life [8], to be more likely to experience weight-based teasing [9], to experience social marginalization [10], and to have poorer levels of academic achievement [11]. Obesity in childhood tends to persist into adulthood [6] where obesity is associated with increased risk of type 2 diabetes mellitus, some types of cancer [12], and premature death [13].

The use of electronic media (EM) such as TV viewing, video gaming and personal computer (PC) use has been found to be a risk factor for higher BMI and for being overweight in a number of studies [14,15]. However, the evidence is somewhat inconsistent across types of EM and across sex [16]. Physical activity is another important determinant of weight status and is associated with increased energy expenditure and lower BMI scores across a number of studies [17]. Several studies have found moderate-to-vigorous physical activity (MVPA) to be weakly correlated with EM behaviors [18,19] meaning that some adolescents may spend a substantial amount of time in both types of behavior. This study aims to investigate these patterns of behavior and their association with body composition as measured by BMI.

EM use and MVPA could potentially be additive or interactive in their associations with BMI. Additive associations would imply that associations between one type of behavior and BMI would be stable irrespective of how much time is spent in the other type of behavior. Practical implications of additive associations would be that the reduction in EM usage would be equally beneficial for individuals across all activity levels. It would also be possible to compensate for the risk associated with high levels of EM use by being physically active. An interactive relationship however, would imply that associations between EM use and BMI would depend upon the level of MVPA.

The relevance of investigating both additive and interactive associations between EM and MVPA is highlighted by a number of studies that have identified subgroups of adolescents reporting relatively high levels of both MVPA and EM usage [20-23]. Importantly, these studies all suggest sex specific patterns in associations where time spent using EM has been found to be an important determinant for weight status among physically active girls, but not among active boys.

National recommendations from USA [24], Canada [25] and Australia [26] suggest that parents should limit the exposure of total media time (USA), recreational screen time (Canada), and electronic media for entertainment (Australia) to a maximum of two hours daily for children and adolescents. In the current study, we explore whether exceeding two hours in one or multiple types of EM is associated with BMI or risk for overweight in adolescents participating in a large scale cross-national survey across Europe and North America. Further, we wish to determine if associations between EM usage and BMI are moderated by being physically active according to current recommendations.

## Methods

### Sample

The data used in this study stem from the 2009/2010 survey of “Health Behaviour in School-aged Children (HBSC) study: A WHO Cross-National Survey” [27]. School or school class was used as the primary sampling unit and samples were stratified to ensure national representativeness. Each participating country followed the same international research protocol to ensure consistency in survey instruments, consent, data collection and processing procedures [28]. The survey was conducted in the classroom administered by the class teacher. Student participation was voluntary, and active, or we sought passive consent from school administrators, parents, and children in accordance with the national requirements of each respective country. Ethics clearance was obtained by each country from a university-based review board or equivalent regulatory body. The response rate is estimated to be over 60% in most countries. More details on the HBSC study procedures can be found elsewhere [28,29].

The population selected for sampling was 11, 13 and 15 year olds attending school with the desired mean age for the three age groups being 11.5, 13.5 and 15. 5 years. We chose to exclude 11 year olds as they had substantially higher proportions of missing responses for BMI. Participants from Armenia, Belgium (Wallonia), England, Greenland, Ireland, Lithuania, Scotland, Wales, and Canada were also excluded due to high proportions of missing responses for BMI (over 20%), or lack of relevant data (Lithuania). Finally, the analysis included 107184 participants (54508 girls and 52676 boys) from 30 countries (see Table 1 for the list of countries).

**Table 1:** **Country and sex stratified sample size, prevalence of overweight and obesity and missing BMI responses**

	**% Overweight/obese**			**% Missing BMI responses**			**Sample**		
	**Boys**	**Girls**	**Total**	**Boys**	**Girls**	**Total**	**Boys**	**Girls**	**Total**
United States	31%	22%	27%	10%	12%	11%	2303	2068	4371
Greece	26%	14%	20%	5%	6%	6%	1645	1660	3305
Slovenia	22%	13%	18%	5%	6%	5%	1850	1783	3633
Croatia	23%	11%	17%	4%	4%	4%	2115	2268	4383
Portugal	20%	14%	17%	6%	6%	6%	1309	1544	2853
Spain	19%	12%	16%	8%	8%	8%	1826	1957	3783
Italy	21%	12%	16%	8%	10%	9%	1618	1634	3252
Poland	19%	12%	16%	4%	5%	5%	1386	1481	2867
Czech Republic	20%	9%	14%	3%	4%	4%	1426	1573	2999
Finland	17%	12%	14%	7%	8%	8%	2116	2262	4378
Hungary	18%	10%	14%	10%	9%	10%	1596	1795	3391
Israel	19%	10%	14%	11%	11%	11%	1168	1502	2670
Iceland	17%	12%	14%	13%	10%	11%	3798	3698	7496
Luxembourg	17%	10%	14%	13%	15%	14%	1558	1591	3149
Romania	19%	9%	14%	9%	8%	9%	1870	1910	3780
Macedonia	19%	8%	13%	13%	15%	14%	1464	1401	2865
Germany	15%	10%	12%	17%	18%	17%	1570	1748	3318
Estonia	15%	9%	12%	17%	17%	17%	1362	1458	2820
Slovakia	17%	7%	12%	11%	10%	11%	1924	1993	3917
Turkey	17%	8%	12%	7%	10%	9%	1819	1943	3762
Belgium-VLG	12%	10%	11%	7%	7%	7%	1346	1333	2679
Latvia	13%	8%	11%	8%	6%	7%	1357	1435	2792
Norway	14%	8%	11%	15%	19%	17%	1349	1314	2663
Switzerland	13%	7%	10%	8%	9%	9%	2448	2387	4835
Sweden	14%	7%	10%	15%	14%	14%	2219	2235	4454
Ukraine	13%	7%	10%	8%	6%	7%	1777	1982	3759
France	11%	7%	9%	19%	20%	20%	1989	2019	4008
Denmark	8%	7%	8%	22%	17%	19%	1341	1431	2772
Russian Federation	11%	6%	8%	11%	12%	11%	1565	1557	3122
Netherlands	8%	6%	7%	19%	16%	17%	1562	1546	3108
Total	18%	11%	14%	11%	11%	11%	52676	54508	107184

Countries are sorted by total prevalence of overweight and obesity.

### Measures and constructs

Self-reported information about height and weight was used to calculate BMI using the formula kg/m2. The questions were “How much do you weigh without clothes?” (in kg) and “How tall are you without shoes?” (in cm). The BMI scores were recoded into standardized z-scores based upon the guidelines from Childhood Obesity Working Group of the International Obesity Taskforce [30]. Calculation of continuous and categorical BMI variables was done by the zanthro command developed by Vidmar and colleagues [31] available for the Stata software (version SE 13.0). The overweight and obese categories corresponding to adult BMI 25–29.99 and 30+ respectively were then collapsed into a dichotomous variable encompassing all individuals with a BMI score corresponding to an adult BMI of 25 or more.

Time spent using EM was assessed through the following items: “About how many hours a day do you usually watch television (including videos) in your free time?”, “About how many hours a day do you play PC-games or TV-games (Playstation, Xbox, GameCube etc.) in your free time?” and “About how many hours per day do you use a PC for chatting online, surfing the internet, writing emails, homework etc. in your free time”. All three questions had the following nine response options: “None at all”, “About half an hour a day”, “About 1 hour a day”, “About 2 hours a day”, “About 3 hours a day”, “About 4 hours a day”, “About 5 hours a day”, “About 6 hours a day”, “About 7 or more hours a day”. Vereecken and colleagues [32] assessed the test-retest reliability of the item measuring hours spent watching TV, and found an intraclass correlations (ICC) of .76 for boys and .54 for girls. The relative/predictive validity of the same item compared with a 7-day TV diary resulted in an ICC of .36 for boys and ICC of .54 for girls, with our questionnaire item, exceeding time reported in the TV diary by approximately one hour per day for boys, and half an hour for girls. Previous studies have observed good test-retest reliability and validity for items similar to the additional screen based items used in our study [33].

EM items were dichotomized in order to test whether exceeding the recommended cut-off of two hours or less for daily EM use was associated with BMI. A binary variable was constructed where responses of two hours or more were coded 1 based on the consideration that they exceeded the daily recommendations of maximum two hours a day of EM. Further, we constructed dummy variables indicating whether individuals exceeded 2 hours or more daily in none, one, two or three types of EM.

The measure of physical activity includes both moderate and vigorous physical activity (MVPA): “Over the past 7 days how many days were you physically active for a total of at least 60 minutes?”. This question was developed by Prochaska and colleagues [34] in a slightly modified version asking in addition “how many days in a typical week”. For this original version good test-retest stability [34,35] and validity in terms of substantial correlations with accelerometers was shown [34,36]. Building on WHO recommendations that 5–17 year old youth should attain 60 minutes of MVPA daily [37] we constructed a dichotomous variable to discriminate between those who reported being physically active seven days per week and those who did not.

Family affluence was assessed by the Family Affluence Scale [38] as a sum score of four items: Does your family own a car, van or truck? (0–2 points). Do you have your own bedroom for yourself? (0–1 points). During the past twelve months, how many times did you travel away on holiday (vacation) with your family? (0–2 points); and how many computers does your family own? (0–2 points).

Breakfast consumption was measured by two questions. Students were asked to estimate how many weekdays they had breakfast (i.e. having more than a glass of milk or fruit juice). Possible response categories were “never”, and 1, 2, 3, 4, or 5 days and how many weekend days they had breakfast with the possible response categories “never”, 1 and 2 days. The responses on two items were added up and recoded into “less than daily” versus “daily”. This variable was used as a proxy for dietary quality as it has been reliably associated with overall quality of diet and with BMI [39,40].

Interaction terms were constructed by multiplying the binary variable representing MVPA according to recommendations with the dummy variables representing exceeding two hours in one, two or three types of EM daily.

### Statistical analysis

Confidence intervals in Table 2 were calculated in Stata (Version 12), adjusting for clustering in the school class (psu) and on the country level. Multilevel modeling was found to be necessary for analysis of this data as the design effect for BMI z-scores was substantial (DEFF = 68.81)^a^. A DEFF greater than 2 suggests that dependency in the data needs to be taken into account during estimation. Consequently, multilevel modeling is highly appropriate for this data as standard errors would be underestimated if we did not account for the hierarchical data structure.

**Table 2:** **Prevalence of electronic media behaviors, physical activity, mean levels of BMI z-scores and prevalence of overweight and obesity across behavioral patterns**

	**Girls**				**Boys**			
	**Prevalence In sample**	**Overweight/obese prevalence within categories**	**Mean BMI z-score**	**95% CI**	**Prevalence In sample**	**Overweight/obese prevalence within categories**	**Mean BMI z-score**	**95% CI**
**Physical activity**								
MVPA < 7 days	89%	11%	−0.08	(−0.09 to −0.07)	79%	19%	0.11	(0.10 to 0.12)
MVPA 7 days	11%	9%	−0.19	(−0.21 to −0.16)	21%	14%	0.01	(−0.01 to 0.03)
**Exceeding 2 hours using EM**								
No EM	23%	9%	−0.18	(−0.20 to −0.16)	19%	15%	0.01	(−0.01 to 0.03)
1 EM	34%	11%	−0.08	(−0.10 to −0.07)	28%	18%	0.10	(0.08 to 0.12)
2 EM	29%	11%	−0.07	(−0.08 to −0.05)	29%	19%	0.10	(0.08 to 0.12)
3 EM	14%	13%	−0.06	(−0.08 to −0.03)	24%	20%	0.12	(0.10 to 0.14)
**Meeting PA guidelines and**								
No EM	3%	6%	−0.32	(−0.37 to −0.27)	5%	13%	−0.03	(−0.08 to 0.01)
1 EM	4%	9%	−0.15	(−0.19 to −0.10)	6%	15%	0.04	(0.00 to 0.08)
2 EM	3%	10%	−0.13	(−0.18 to −0.08)	5%	14%	0.01	(−0.03 to 0.05)
3 EM	1%	10%	−0.14	(−0.22 to −0.06)	5%	15%	0.01	(−0.03 to 0.06)
**Not meeting PA guidelines and**								
No EM	20%	9%	−0.16	(−0.18 to −0.14)	14%	16%	0.03	(0.00 to 0.05)
1 EM	31%	11%	−0.07	(−0.09 to −0.06)	23%	19%	0.11	(0.09 to 0.13)
2 EM	26%	11%	−0.06	(−0.07 to −0.04)	23%	20%	0.13	(0.10 to 0.15)
3 EM	12%	13%	−0.05	(−0.07 to −0.02)	19%	21%	0.15	(0.12 to 0.17)
**Total**		11%	−0.10	(−0.11 to −0.09)		18%	0.09	(0.07 to 0.10)

Confidence intervals account for dependency in data at the school class and on the country level.

The multilevel regression models are estimated with the complex random command in Mplus (version 7.0) [41]. Only the fixed effects part of the regression is presented, but the country level variance in the outcome variables was estimated and the standard errors of the class level (PSU) were accounted for in the statistical models. The presented regression tables can thus be interpreted as a regular regression model where the intercept is allowed to vary across both country and class level^b^.

The linear regression coefficients presented are unstandardized and can be interpreted as the estimated difference in BMI z-scores from the reference group associated with the different independent variables.

Regression models in Table 3 were estimated with three interaction terms between the dichotomous PA item and a dummy indicator for exceeding 2 hours in 1, 2 or 3 different types of EM behavior. The *model constraint* command in Mplus (version 7.0) [41] was used to calculate estimates and standard errors for the predicted difference in BMI and odds compared to the reference group. Consequently all estimates should be interpreted as predicted change in BMI z-scores or odds for overweight compared to the reference group (high PA and spending 2 hours or less daily using any type of EM).

**Table 3:** **Random intercept regression models showing predicted change in BMI z-scores and odds ratio for being overweight or obese for failing to meet PA guidelines and exceeding 2 hours using 1, 2 or 3 types of EM and for meeting PA guidelines and exceeding 2 hours using 1, 2 or 3 types of EM**

	**Girls**				**Boys**			
	**b**	**95% CI**	**OR**	**95% CI**	**b**	**95% CI**	**OR**	**95% CI**
**Not meeting MVPA recommendations and**								
1 EM	**0.07**	**(0.04 to 0.11)**	**1.20**	**(1.08 to 1.35)**	**0.07**	**(0.02 to 0.11)**	**1.23**	**(1.08 to 1.40)**
2 EM	**0.08**	**(0.04to 0.12)**	**1.29**	**(1.12 to 1.48)**	**0.08**	**(0.02 to 0.14)**	**1.32**	**(1.12 to 1.55)**
3 EM	**0.09**	**(0.04 to 0.14)**	**1.45**	**(1.25 to 1.69)**	**0.09**	**(0.03 to 0.15)**	**1.38**	**(1.16 to 1.64)**
**Meeting MVPA recommendations and**								
1 EM	**0.17**	**(0.09 to 0.26)**	**1.57**	**(1.17 to 2.10)**	0.06	(−0.01 to 0.13)	1.05	(0.92 to 1.21)
2 EM	**0.18**	**(0.09 to 0.26)**	**1.66**	**(1.24 to 2.22)**	0.04	(−0.04 to 0.11)	1.02	(0.87 to 1.20)
3 EM	**0.15**	**(0.06 to 0.23)**	**1.73**	**(1.25 to 2.38)**	0.05	(−0.04 to 0.14)	1.14	(0.94 to 1.38)

The reference group is physically active adolescents who spend less 2 hours or less using EM daily.

Estimates are adjusted for family affluence and daily breakfast consumption.

Significant estimates are in bold.

In light of established sex differences in the associations between health behaviors and weight status [23,42,43], we tested two-way interactions between sex and EM as well as MVPA using both continuous BMI z-scores in a random intercept linear regression models, and with overweight as a dichotomous outcome in a random intercept logistic regression model. All interactions were statistically significant (p < .029) indicating that sex stratification of analyses was appropriate. All regression models were controlled for family affluence (centered at grand mean) and daily breakfast consumption as these are potential confounders.

## Results

Table 1 shows descriptive statistics across the countries including the sample size, % overweight and % missing BMI responses. For overweight, the prevalence ranged from 27% in the US to 7% in the Netherlands. In all countries there was a higher prevalence of overweight among boys. The proportion of missing BMI responses ranged from 4% in Croatia to 20% in France with no consistent sex differences in the prevalence of missing responses.

Table 2 shows the prevalence of MVPA, EM use and the combination of the two among boys and girls. It also shows mean levels of standardized BMI z-scores and prevalence of overweight across the different behavioral patterns. Overall, 18% of boys and 11% of girls were classified as overweight. The mean standardized BMI scores were -.010 for girls and 0.09 for boys, suggesting that girls in this sample had somewhat lower BMI scores relative to the WHO standardized values for this age group. Boys in the sample were found to have higher BMI scores than the WHO standard. A total of 21% of boys reported meeting MVPA guidelines compared to 11% of girls. The prevalence of overweight and mean BMI was lower among physically active boys and girls^c^. The use of EM varied by sex, with usage of multiple types of EM being more common among boys. More girls than boys reported spending two hours or less daily in any type of EM (23% girls vs. 19% boys), or exceeding 2 hours in just one type of EM behavior (34% vs. 28%, respectively). Boys were more likely than girls to exceed 2 hours in all three types of EM behaviors daily (24% boys, and 14% girls). A trend for higher BMI and overweight prevalence was found across increasing number of EM behaviors for both sexes. Crude random intercept regression models estimating linear coefficients and odds ratios supported the trends for significant higher odds for overweight and BMI z-scores among individuals exceeding 2 hours in 1, 2 or 3 EM behaviors. The combination of PA with any kind of EM use was less prevalent among girls than among boys. Both the prevalence of overweight and mean BMI were lower among physically active adolescents who spent 2 hours or less daily in any type of EM compared with active adolescents who reported exceeding 2 hours using one or more types of EM^c^.

Table 3 shows the fixed effects estimates from random intercept regression models estimating the predicted change in BMI z-scores and odds for being overweight when exceeding two hours daily in one, two or three types of EM adjusted for family affluence and breakfast consumption. Associations are also shown both for those who report meeting the MVPA recommendations, and for those who do not. The reference group is physically active adolescents who spent 2 hours or less in any type of EM. Exceeding 2 hours daily in one or more EM behaviors was associated with higher BMI scores and higher odds for overweight among both boys and girls who reported not meeting MVPA guidelines. However, we found substantial sex differences for those who reported meeting MVPA recommendations. None of the EM variables were found to be significantly associated with BMI or odds for overweight among physically active boys. In contrast, among physically active girls, EM use was found to be positively associated with both BMI and risk for overweight.

We found significant interactive associations for the linear models regressing BMI on EM and MVPA for girls. These were found between meeting guidelines of MVPA and exceeding one (b = −0.10, p < .014) and two (b = −0.10, p < .012) EM behaviors daily. These indicate that associations between BMI and exceeding two hours in two or three types of EM behaviors daily are on average −0.10 standardized BMI points lower for those who reported not meeting MVPA guidelines relative to those who did.

For boys we found significant interactions between meeting MVPA guidelines and exceeding two hours in two (logit = .25, p < .014) and three (logit = .19, p < .023) EM behaviors daily. This implies that the odds for being overweight when exceeding two hours in two and three types of EM daily were significantly lower for those who reported meeting MVPA guidelines versus those who did not. Although estimates for all combinations of MVPA and EM behaviors are presented, only the above mentioned ones were significantly different from each other.

Table 4 shows estimates of associations between spending 2 hours or more in EM behaviors and failing to meet MVPA recommendations with BMI z-scores and odds ratios for being overweight. All EM behaviors and failing to meet MVPA recommendations were consistently associated with higher odds for being overweight and for higher BMI z-scores among both boys and girls.

**Table 4:** **Linear and logistic random intercept models showing crude estimates of association between risk factors and BMI z-scores and odds ratios for being overweight/obese**

	**Girls**				**Boys**			
	**B**	**95% CI**	**OR**	**95% CI**	**B**	**95% CI**	**OR**	**95% CI**
2 hr TV	0.08	(0.06 to 0.10)	1.38	(1.30 to 1.48)	0.06	(0.04 to 0.08)	1.20	(1.14 to 1.27)
2 hr PC	0.09	(0.07 to 0.11)	1.14	(1.07 to 1.21)	0.07	(0.05 to 0.09)	1.15	(1.10 to 1.22)
2 hr Gaming	0.06	(0.03 to 0.08)	1.29	(1.20 to 1.39)	0.04	(0.02 to 0.06)	1.18	(1.12 to 1.25)
No daily MVPA	0.12	(0.09 to 0.15)	1.29	(1.16 to 1.43)	0.14	(0.11 to 0.16)	1.50	(1.41 to 1.61)

The reference group is physically active adolescents who spend less 2 hours or less using any of the EM behaviors daily.

Estimates are controlled for family affluence status and daily breakfast consumption.

Additional analyses showed that neither interactions between each respective type of EM behavior, nor a cumulative measure of EM time were found to be significantly in predicting odds for overweight or BMI z-scores. Exceeding 2 hours of cumulative EM use was associated with an increase in BMI z-score of 0.13 (0.08 to 0.17) for boys and 0.13 (0.10 to 0.16) for girls. Odds of overweight were 1.24 (1.11 to 1.39) for boys and 1.41 (1.25 to 1.58) for girls who exceeded 2 hours in cumulative EM use.

## Discussion

The main findings of this study indicate that spending in excess of 2 hours daily in any EM behavior, or failing to meet recommendations for daily MVPA are associated with increased odds for overweight. Associations between EM use and overweight differed across sex and across level of physical activity as EM use was found to be associated with increased BMI and risk of overweight among physically active girls, but not among physically active boys.

An important finding of this study indicates that meeting MVPA recommendations was found to moderate the association between EM behaviors and BMI (girls) and overweight status (boys). Consequently associations between BMI and exceeding 2 hours daily in EM behaviors differs between adolescents who report to be sufficiently active according to international recommendations [37] and those who report being less physically active. Specifically, we found that exceeding 2 hours daily in one or more EM behaviors was associated with higher BMI scores and risk for overweight among both boys and girls who did not meet the MVPA recommendations. Among the physically active adolescents we found clear and consistent sex differences indicating that exceeding 2 hours in one or more types of EM behaviors is indeed associated with BMI and risk for overweight among girls, but not among boys. This also corresponds with previous reports suggesting that the use of EM is associated with weight status among physically active females, but not among males [20,21,23]. Other studies confirm this pattern in adult populations [44,45].

In contrast, studies by Ekelund and colleagues [46] and Chaput and colleagues [47] failed to identify interactions between accelerometer measured MVPA and sedentary time in relation to waist circumference and a range of cardio-metabolic outcomes. While these studies use data of superior quality relative to ours, we speculate that the difference in results partly may be due to sex differences in these interactions. Our results suggest interactions in opposite directions between the sexes and we anticipate that these may cancel each other out when analyzing the sexes together.^d^ Another possible explanation for the difference in the findings is that there indeed is no dose–response relationship in the interactions, but that the interactive associations are nonlinear.

There are a number of possible explanations for our findings. First, it is possible that there are substantial sex differences in the types and intensity of MVPA performed by the different sexes. This is plausible as a number of studies have found boys to perform more vigorous physical activity than girls [48,49]. Further, it is also important to consider the sex differences in development through puberty. As adolescent boys are likely to have a greater muscle mass compared to girls, they will also use more calories than girls when being physically active [50]. Thus, our results appear to be in support of other studies that have concluded PA to be more important for the body composition of boys relative to girls [51]. Finally, it is also possible that boys and girls choose different behavioral patterns, or are encouraged to do different behaviors based upon their weight status.

Sex differences in the prevalence of the behavioral patterns also correspond with other studies that have found it to be more common for boys to spend a relatively high amount of time in both physical activity and screen behaviors [18,20,23].

Although it is beyond the scope of the current study to determine the mechanisms which may explain the current results, we speculate that our findings cannot be explained by the different energy expenditures associated with MVPA and EM behaviors. Rather, evidence from physiological studies indicate that MVPA and sitting elicit qualitatively different biological responses that may thus influence both weight regulation and overall health risk through different mechanisms than energy expenditure alone [52].

Our results are in line with previous studies suggesting that daily MVPA is associated with reduced risk of overweight [42,53]. We also found that exceeding 2 hours in each of the specific EM behaviors was associated with increased odds of overweight. For TV viewing, this corresponds well with the literature as a comprehensive review on the topic [15] concluded that there is a dose–response relationship between TV viewing and BMI and that individuals who spend more than 2 hours watching TV are at increased risk for overweight or obesity. Our findings of increased odds for overweight among boys and girls for gaming and for PC use among boys contributes to a body of mixed evidence as indicated by the review of Rey Lopez and colleagues [16].

Prevalence of overweight varied greatly across countries in similar patterns as seen in previous studies on youth [42,54] and younger children [55]. The highest prevalence levels were found in the USA and in the Mediterranean countries while the lowest prevalence rates were generally found in Northern and Eastern Europe. It should be mentioned that many of the countries with high numbers of overweight youth (e.g. countries in the UK) were not included in the analysis due to high numbers of missing data.

Limitations include the cross-sectional design of the study, limiting causal interpretations of the results. This implies that the associations may be equally likely to result from the tendency of adolescents of different weight status to prefer specific behavioral patterns. Indeed, a 2 year longitudinal study of adolescents found neither MVPA nor sedentary time to be prospectively associated with waist circumference, but found waist circumference to predict sedentary time at follow up [46].

Another limitation is the use of self-reported height and weight as young people tend to under-report weight [56,57] and over-report height [56,58]. However, self-reported height and weight has been found to be useful in epidemiological studies as it correlates highly with actual weight and has been found to be reliable for the prediction of obesity related morbidities and behaviors [59].

The MVPA measurement used is relatively crude both in terms of dose and intensity of activity suggesting that there is some discrepancy between our estimated prevalence of meeting physical activity guidelines and estimates from studies based upon accelerometer measurement [60]. Nevertheless, a recent review lends support to the MVPA measure for surveillance purposes in large scale studies [61].

Finally, our measures of EM behaviors do not allow for the assessment of multi-tasking. Estimates of total time spent in EM behaviors are therefore likely to have reduced reliability, particularly for individuals who regularly do several of the EM behaviors simultaneously. Nevertheless, the current results indicate that these measurements indeed have some merit as risk factors for weight status among adolescents.

This study also has a number of important strengths including a large sample size with high levels of statistical power, representative samples of adolescents from 30 countries across two continents, and a standardized protocol for data collection ensuring internationally comparable data.

### Implications and future research

The current results indicate that interventions designed to reduce bodyweight should include efforts aiming to reduce recreational use of screen behaviors or other activities involving long periods of uninterrupted sitting in addition to the more common physical activity and diet interventions. The very distinct sex differences in the risk for overweight among physically active adolescents indicate that some interventions may benefit from sex specific adaptations.

Future studies should aim to find out whether the current results may be attributed to the time spent in sedentary behavior and in MVPA, or to qualities specifically associated with screen behaviors that explain the current results. We also encourage future studies to look into the explanations for why our and other studies [20,23,45] find such distinct sex differences associations between EM use and physiological outcomes.

## Conclusions

Exceeding two hours daily using one or more types of EM behavior was found to be associated with increased risk of being overweight, but this association differed for boys and girls and by level of physical activity. While the usage of EM appear to be inconsequential for BMI and the risk of overweight among physically active boys, we find evidence indicating that EM use is associated with BMI and risk for overweight among girls, including those who report complying with MVPA guidelines.

### Endnotes

^a^DEFF = 1 + (average cluster size - 1)*intraclass correlation. Thus 1 + (1739.84-1)*0.039.

^b^Output from the random part of the model can be attained by contacting the first author.

^c^Relative to exceeding 2 hours in no EM behaviors.

^d^Additional analyses revealed that no interactions were significant in our data when sexes were analyzed together.
